# Supplementary material for: SARS-CoV-2 structure and replication characterized by in situ cryo-electron tomography
Source: Nat Commun. 2020 Nov 18;11:5885. doi: 10.1038/s41467-020-19619-7 (PMC7676268; doi:10.1038/s41467-020-19619-7)
Supplement: Supplementary file 1 — Supplementary Information [file 41467_2020_19619_MOESM1_ESM.pdf]

Supplementary Information

SARS-CoV-2 structure and replication characterized by in situ cryo-electron tomography

Steffen Klein[#], Mirko Cortese[#], Sophie L. Winter[#], Moritz Wachsmuth-Melm, Christopher J. Neufeldt, Berati Cerikan, Megan L. Stanifer, Steeve Boulant, Ralf Bartenschlager[*], Petr Chlanda[*]

[*] Correspondence to: petr.chlanda@bioquant.uni-heidelberg.de, ralf.bartenschlager@med.uni-heidelberg.de

[#] Equal contribution

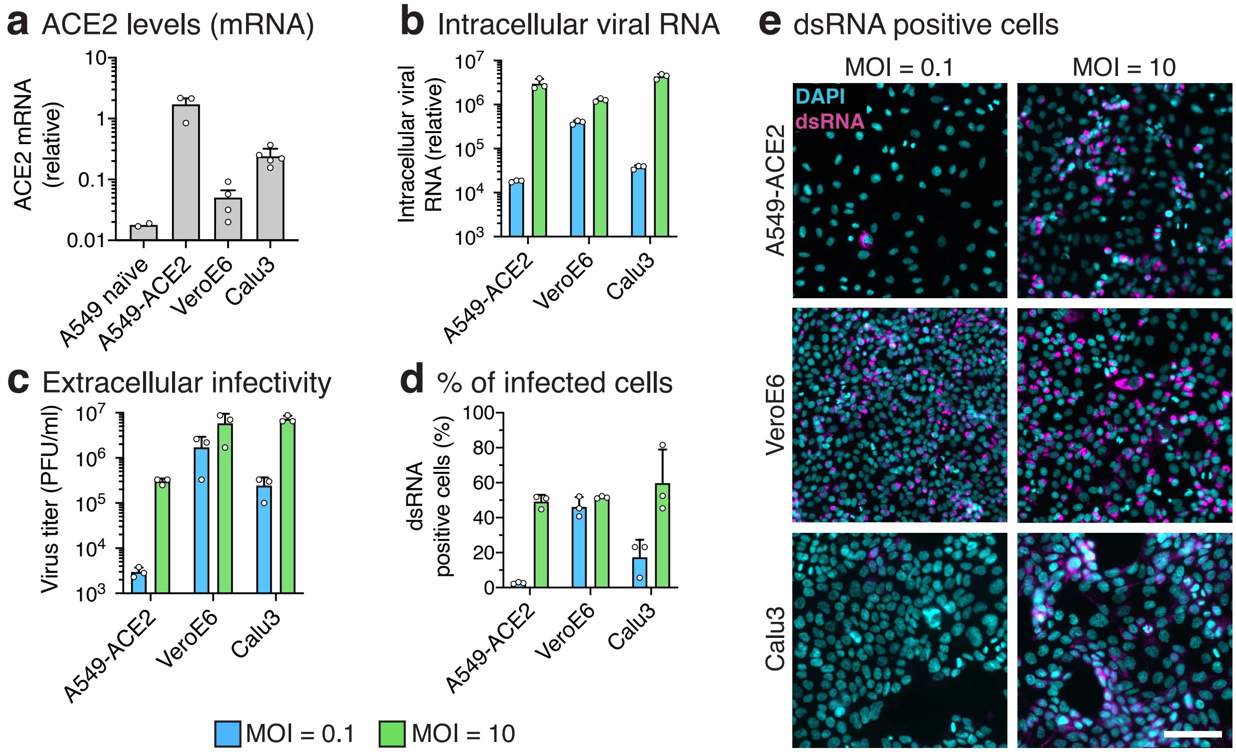

Supplementary Fig. 1 ACE2 mRNA levels and SARS-CoV-2 infection in A549-ACE2, VeroE6 and Calu3 cells. Data are shown as bar plots showing the mean with indicated standard deviation and all data points. **a** ACE2 mRNA levels normalized to HPRT mRNA levels of the three cell lines used in this study determined by RT-qPCR (n = 2 for A549 naïve, n = 3 for A549-ACE2 and n = 4 for VeroE6 and Calu3). **b** Intracellular viral RNA levels (normalized to HPRT mRNA) of the three cell lines infected with SARS-CoV-2 at 16hpi with MOI 0.1 (blue) or 10 (green) determined by RT-qPCR (n = 3). **c** Released extracellular virus measured as plaque forming units (PFU) per ml for the three cell lines infected with SARS-CoV-2 at 16hpi with MOI 0.1 (blue) or 10 (green) (n = 3). **d** Percentages of dsRNA positive cells for the three cell lines infected with SARS-CoV-2 at 16 hpi with MOI 0.1 (blue) or 10 (green) (n = 3). **e** Wide-field fluorescent light microscopy images used for analysis in (d) showing DAPI stained cell nuclei (cyan) and staining with an anti-dsRNA antibody (magenta) in the three cell lines infected with SARS-CoV-2 16 hpi with MOI 0.1 and 10. Scale bar: 100 µm.

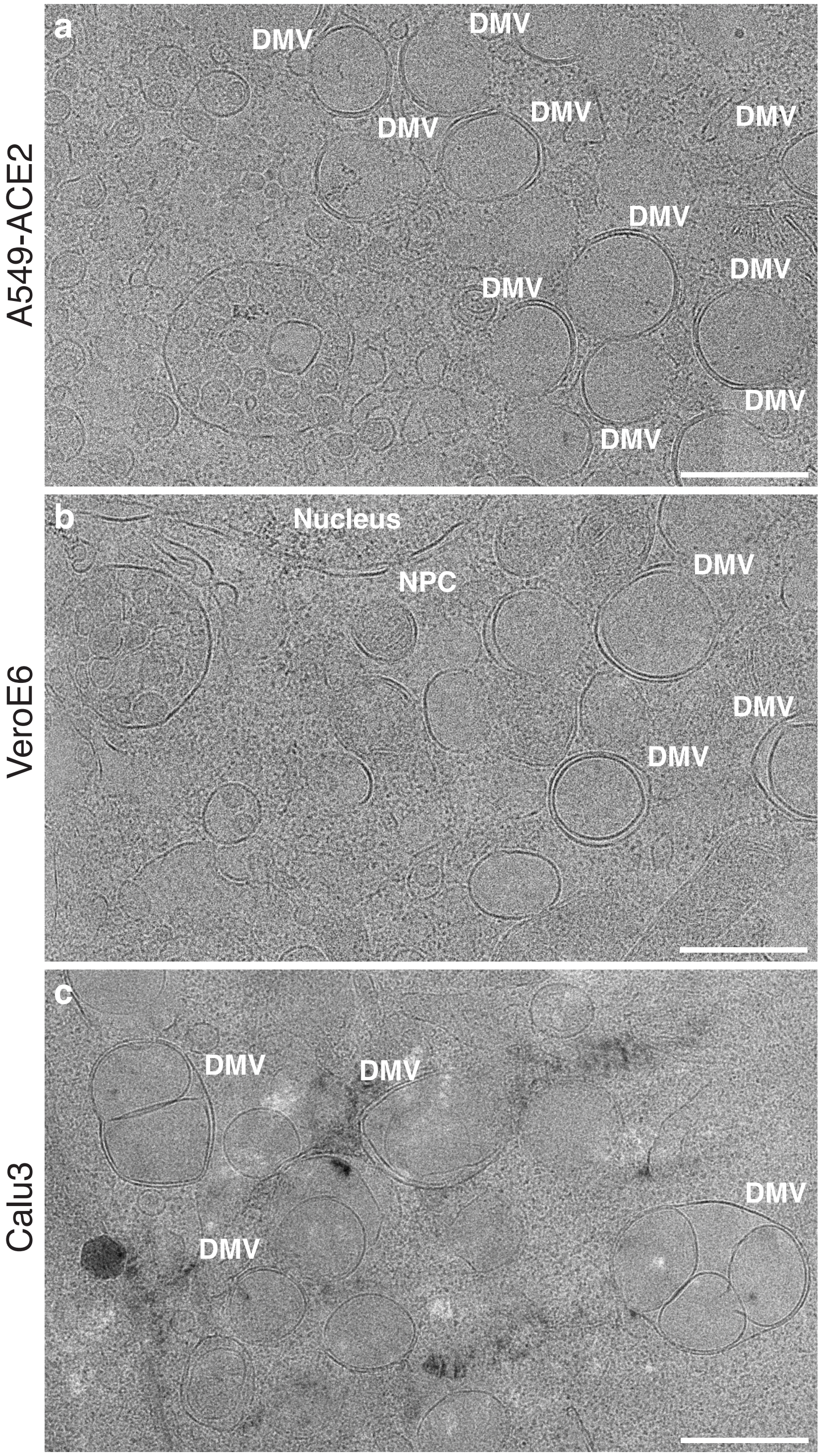

Supplementary Fig. 2 Cryo-EM of DMVs in A549-ACE2, VeroE6 and Calu3 cells. Exemplary images from cryo-EM montages on milled A549-ACE2 (**a**), VeroE6 (**b**) and Calu3 (**c**) cells that were used for tomogram acquisition of DMVs and fused DMVs in Calu3 cells. Due to the thickness of Calu3 and increased clustering of cells, Calu3 cryo-lamellae samples showed partially vitrified regions more frequently. Scale bars: (a–c) 500 nm.

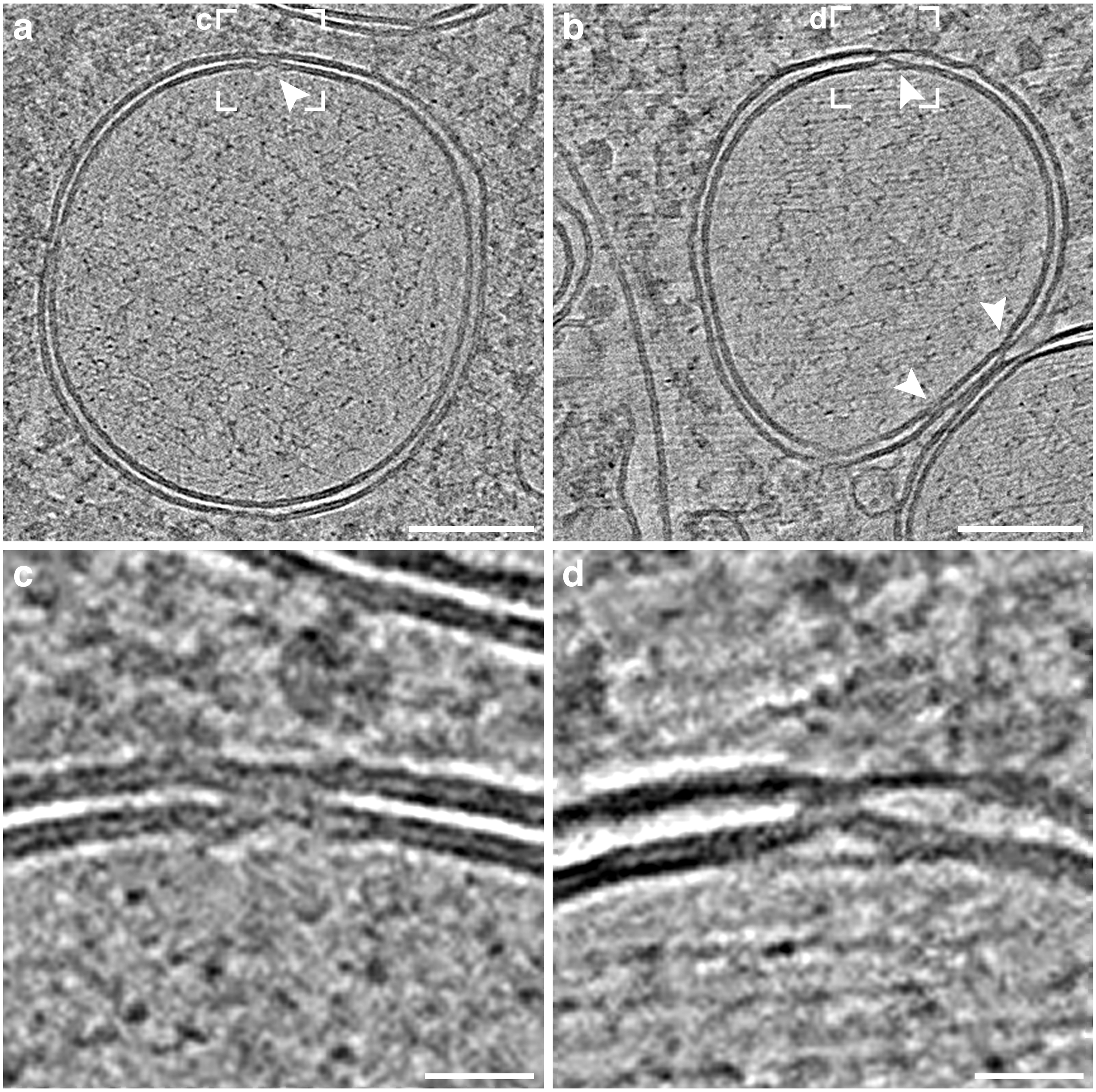

Supplementary Fig. 3 Putative pore complexes clamping the inner and outer DMV membrane and connecting the DMV lumen with the cytoplasm. a, b Slices of tomograms showing DMVs with sites of putative pores (arrowheads), spanning both membranes of the DMV. **c, d** Magnified images of the putative pore complex. Scale bars: (a, b) 100 nm, (c, d) 20nm

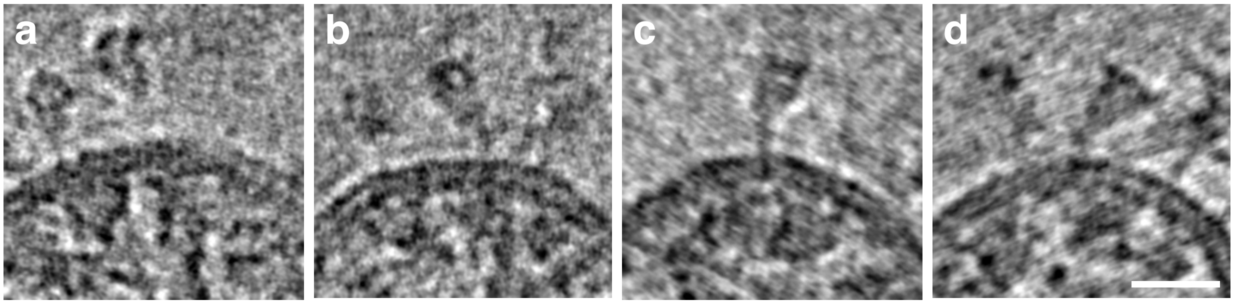

Supplementary Fig. 4 Tilted spike glycoproteins of intracellular virions. Slices of tomograms showing individual S trimers with different tilting angles relative to the viral envelope of 31° (a), 35° (b), 37° (c) and 41° (d). 20 tomogram slices were average and low pass filtered (2 pixel). Scale bar: 20 nm.

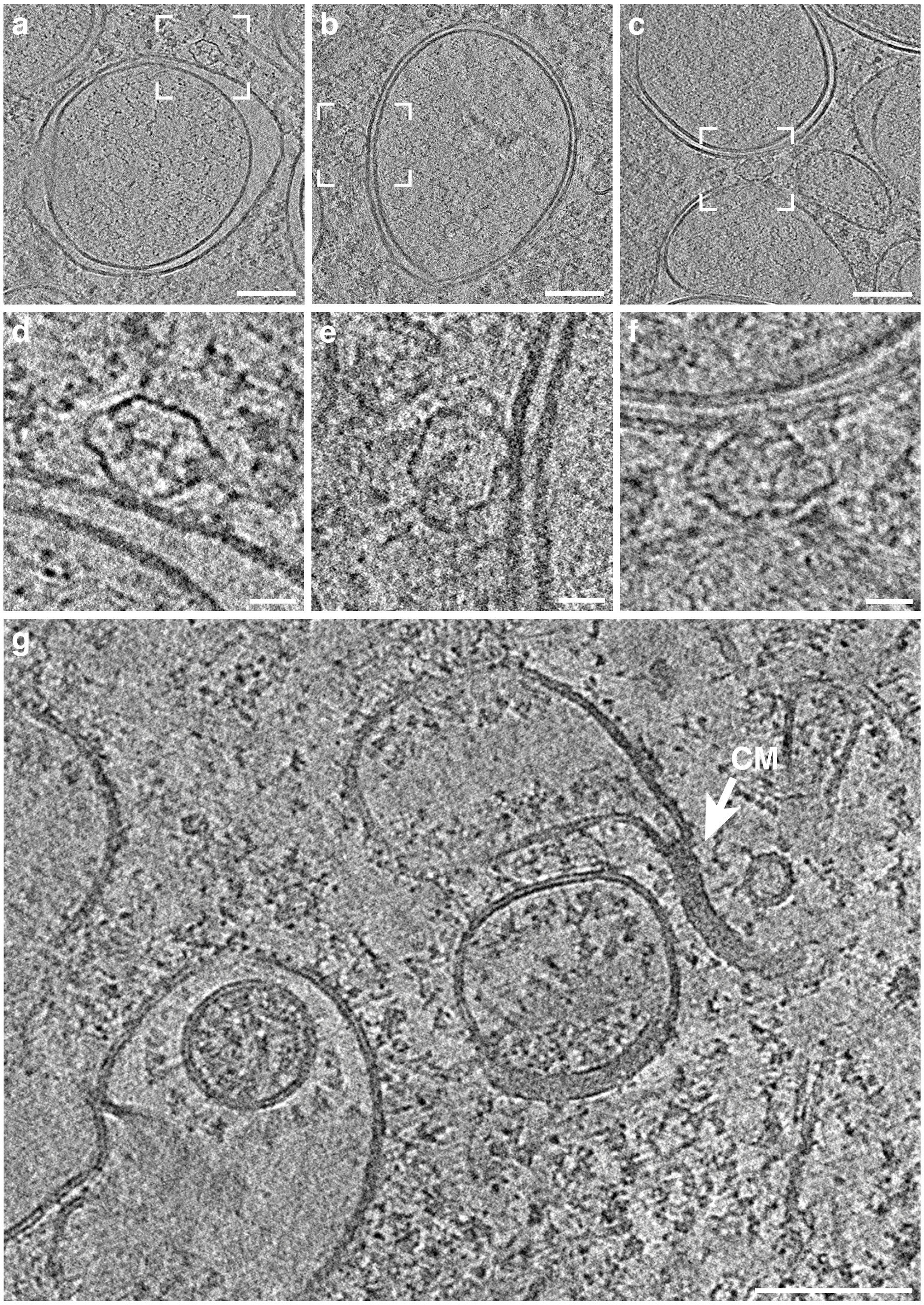

Supplementary Fig. 5 Vault protein complex in the proximity of DMV and putative convolute membrane. a–f Slices of tomograms (average of 10 slices) showing 3 examples of vault complexes in the proximity of the DMV outer membrane found in SARS-CoV-2 infected VeroE6 (a, c, d, f) and in A549-ACE2 cells (b, e). vault complexes are oriented with the longitudinal axis along the outer membrane in all observed cases. In one case a vault complex was found to be located between two DMVs (c, f). **g** Slice of a tomogram of SARS-CoV-2 infected Calu3 cell showing a putative zippered ER cisterna (CM; convoluted membrane) characteristic of a density found inside the cisternae. The diameter of the CM is approximately 18 nm. Scale bars: (a, b, c, d, g) 100 nm, (d, e, f) 20 nm.

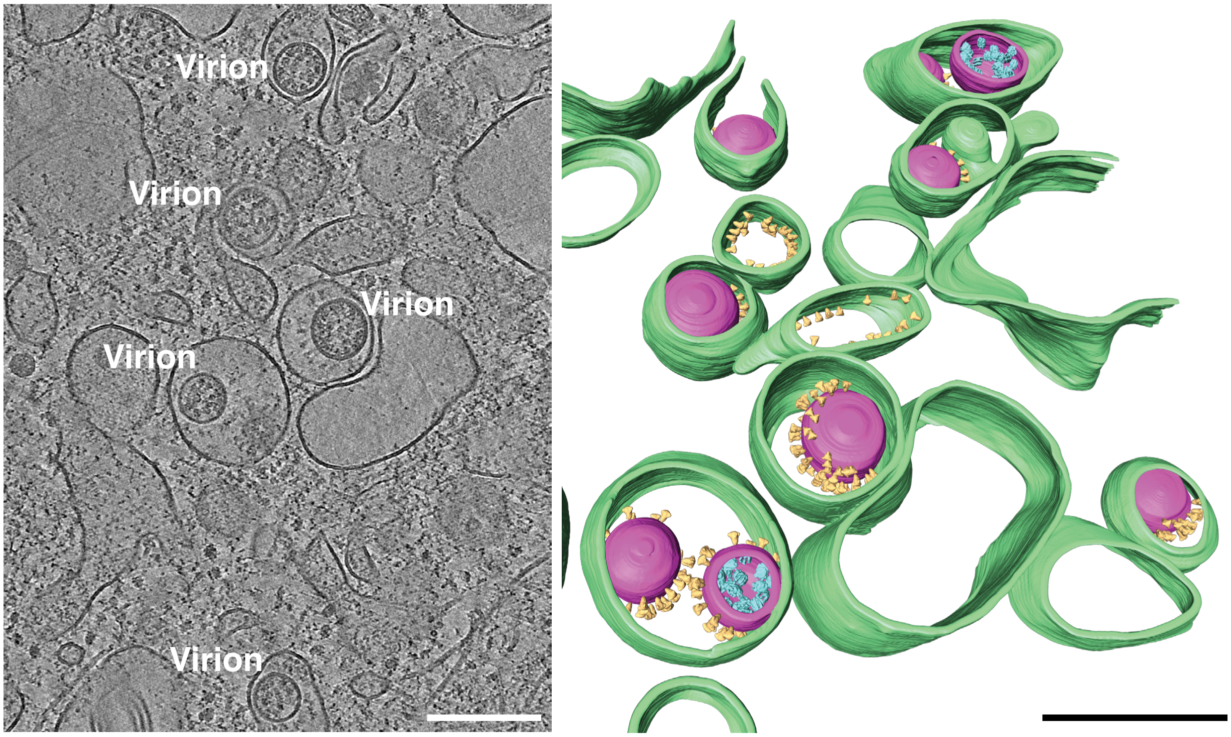

Supplementary Fig. 6 Assembled intracellular virions. Tomogram showing a VeroE6 cell infected with SARS-CoV-2 at 16 hpi. 20 slices of the tomogram were averaged, and a non-local means filter was applied. Intracellular released virions inside the ERGIC lumen are indicated. A 3D volume rendering is shown with cellular membranes (green) and viral membranes (magenta). For each S glycoprotein (yellow) and vRNP (cyan) present in the tomogram the subtomogram average of S glycoprotein was placed in the volume rendering. The S trimer average orientations represent the data in the tomogram, whereas the vRNP average orientations were randomized (Supplementary Movie 3). Scale bar: 200 nm.

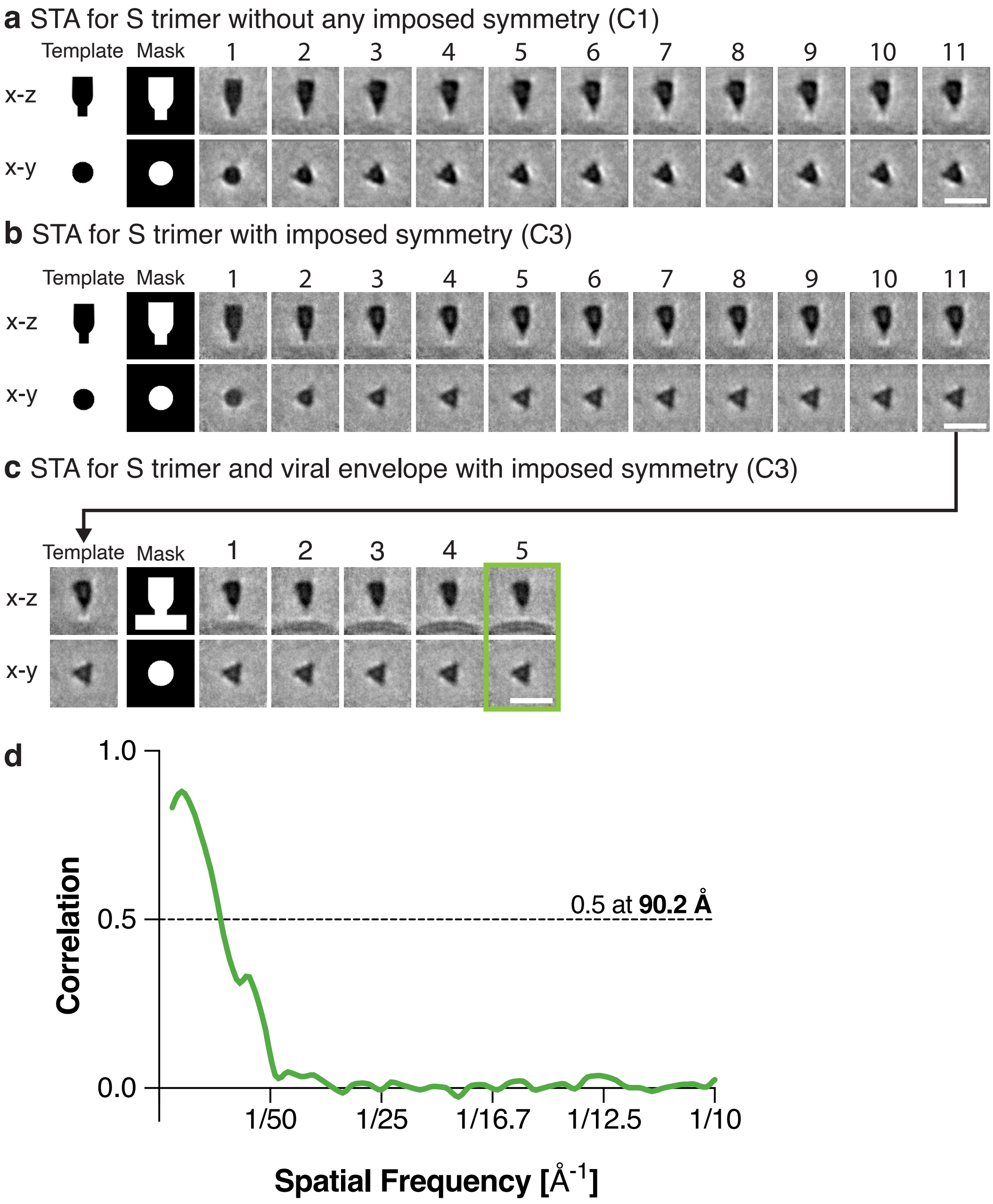

Supplementary Fig. 7 Overview of S trimer subtomogram averaging. Particles (n = 219) corresponding to positions of S trimers in tomograms acquired on cryo-lamella of SARS-CoV-2 infected VeroE6 cells were cropped (160 x 160 x 160 voxels) in Dynamo using the 'general' model. **a** Particles were iteratively aligned and averaged using a template and mask shown on the left without imposing any symmetry. **b** Particles were iteratively aligned and averaged using a template and mask shown on the left with c3 symmetry imposed. **c** Particles were iteratively aligned and averaged using the final average obtained in (b) as a template and a mask shown on the left with c3 symmetry imposed. The final average in B and the final average (green) were c3-symmetrized and used for model fitting in ChimeraX (Supplementary Movie 4). **d** Fourier Shell Correlation analysis of the spike trimer. Scale bars: 10 nm.

a

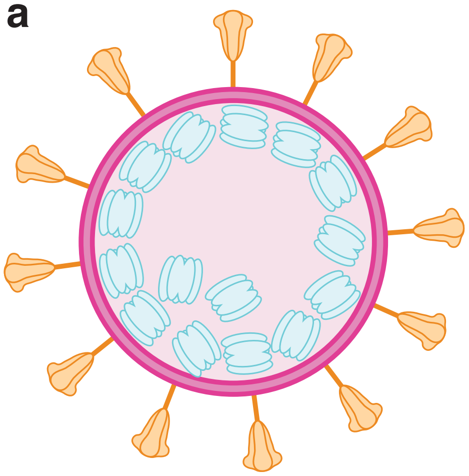

b

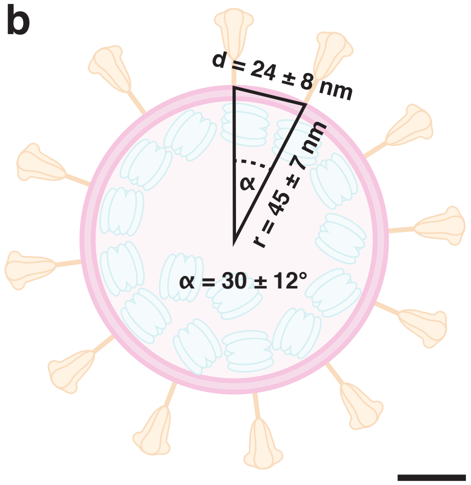

c Central angle

$$\alpha = cos^{-1}\left(1 - \frac{d^2}{2r^2}\right)$$

Error central angle

$$\alpha_{err} = \sqrt{\frac{1}{r^2}\left(1 - \frac{d^2}{4r^2}\right)^{-1} d_{err}^2 + \frac{d^2}{r^4}\left(1 - \frac{d^2}{4r^2}\right)^{-1} r_{err}^2}$$

d

Central angle	Number of points	
43.71	23	
43.69	24	
**41.63**	**25**	$\bar{\alpha} + SD = 42.1°$
41.04	26	
40.68	27	
39.36	28	
38.71	29	
38.60	30	
(…)		
32.09	43	
31.98	44	
31.32	45	
30.96	46	
30.78	47	
**30.76**	**48**	$\bar{\alpha} = 30.4°$
29.92	49	
29.75	50	
29.37	51	
29.19	52	
28.81	53	
(…)		
19.07	122	
19.01	123	
18.95	124	
18.84	125	
18.78	126	
**18.69**	**127**	$\bar{\alpha} - SD = 18.7°$
18.63	128	
18.56	129	

Supplementary Fig. 8 Model of SARS-CoV-2 with estimation of S trimers per virion. a, b Schematic model of a SARS-CoV-2 virion showing the viral envelope in magenta, vRNP in cyan and S trimers in yellow drawn to scale. The two structural transmembrane proteins M and E are not shown. The average radius r = 45 ± 7 nm, average minimum distance between nearest-neighboring S trimers d = 24 ± 8 nm and the corresponding average central angle α = 30 ± 12° are indicated in (b). **c** Equations used for calculations of the central angle α and its error propagation. **d** Table of estimates of the 'Tammes Problem' for different numbers of points on a sphere and the corresponding central angle. The average central angle of 30.4° corresponds closest to a conformation with 48 points on a sphere. For the limits of the confidence interval for the central angle of 18.7° and 42.1° (highlighted in bold), closest matching conformations are 25 and 127 points on a sphere, respectively. Scale bar: 20 nm.

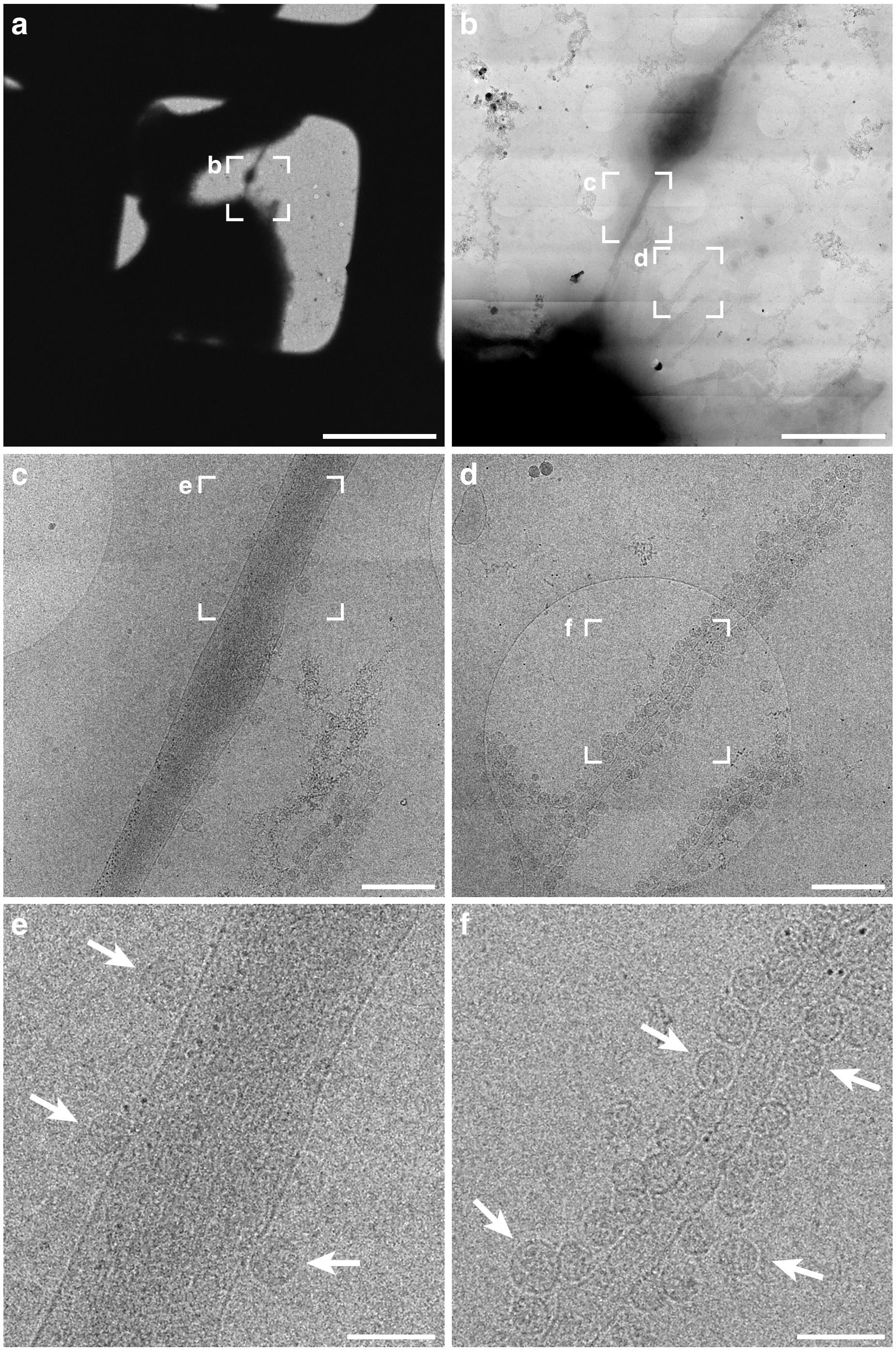

Supplementary Fig. 9 Cryo-TEM of extracellular virions attached on VeroE6 cell protrusions. a Low magnification cryo-TEM image of a 200 Mesh Ti grid coated with a holey carbon (Quantifoil R2/2) with infected VeroE6 cells located in a grid square. **b** Magnified image of cell periphery denoted in (a) by a dashed square. **c, d** Magnified images of cell filopodia decorated with extracellular virions denoted in (b) by dashed squares. **e, f** Magnified images with individual virions (arrows) attached to the filopodia denoted in (c, d) by dashed squares. Scale bars: (a) 50 µm, (b) 5 µm, (c, d) 500 nm, (e, f) 200 nm.

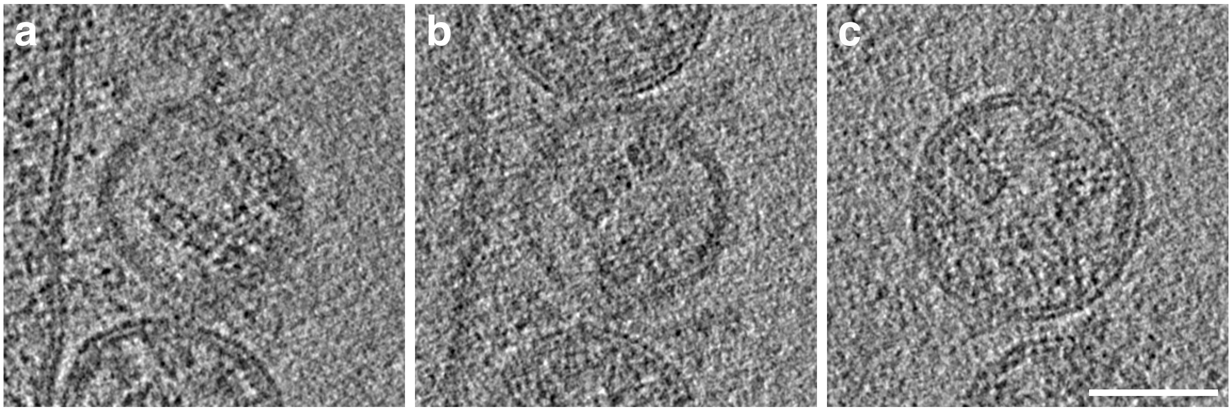

Supplementary Fig. 10 Organization of vRNPs in released extracellular virions. a–c Slices of tomograms of SARS-CoV-2 virions released from VeroE6 cells. Exemplarily, virions displaying linearly arranged vRNPs are shown from 3 tomograms. For better visualization, 6 slices were averaged from 3x binned and low pass filtered (2 pixel) tomograms. Scale bar: 50 nm.

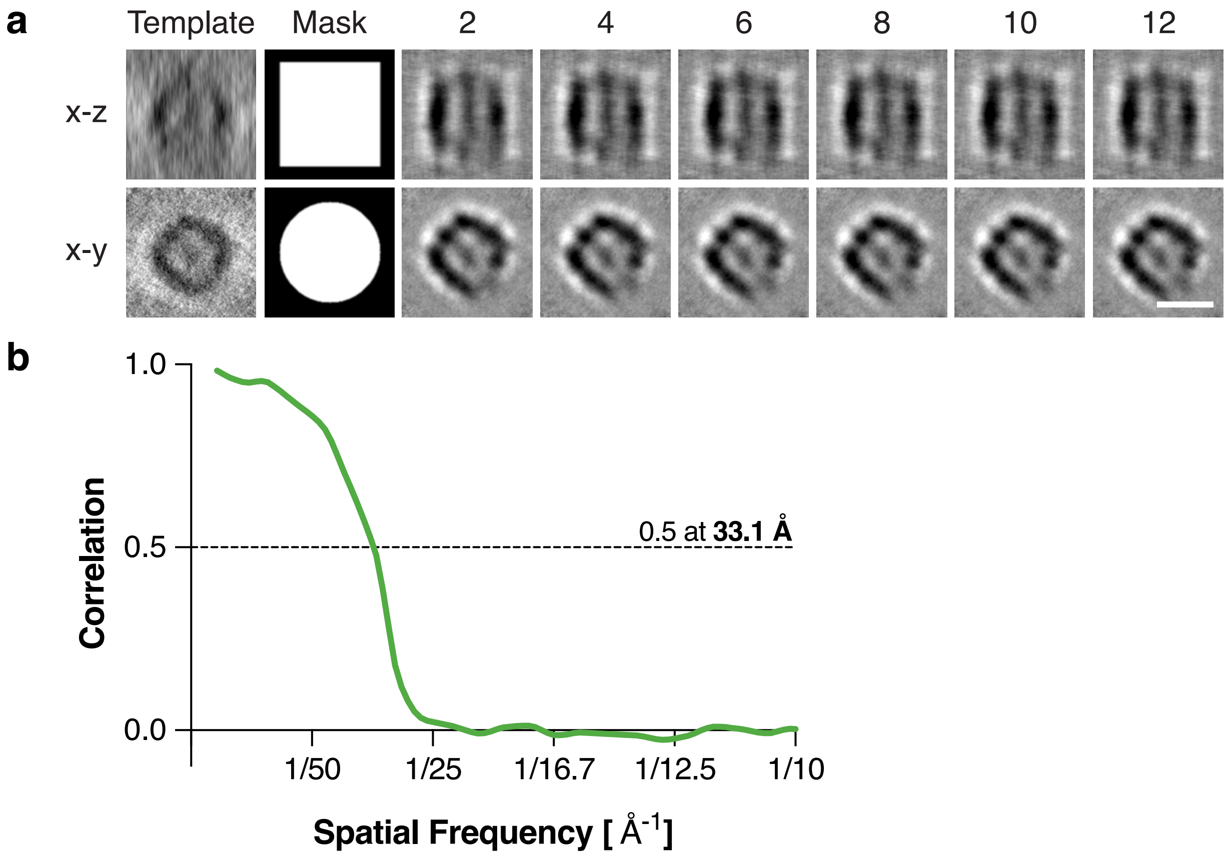

Supplementary Fig. 11 Overview of vRNP subtomogram averaging. a Particles (n = 1570) corresponding to positions of vRNPs in tomograms acquired on cryo-lamella of SARS-CoV-2 infected VeroE6 cells were cropped (168 x 168 x 168 voxels) in Dynamo using the 'general' model. 200 of randomly oriented particles were averaged to generate an initial template. Subsequently, particles were iteratively aligned and averaged using the template and a cylindrical mask (Supplementary Movie 5). Scale bar: 10 nm. **b** Fourier Shell Correlation analysis of the vRNP.

Supplementary Table 1 Sample preparation overview for on-lamella cryo-ET. Summary of biological replicas, number of milled lamellas, number of acquired and analyzed tomograms from each cell line used in the study.

Infection and plunging	Cells	MOI	Infection hpi	Number of lamellae	Number of tomograms	Number of tomograms used for analysis
Sample preparation I	A459-ACE2	7	16	6	11	7
	A459-ACE2	20	16	6	10	2
	A459-ACE2	7	16	7	18	7
Sample preparation II	Calu3	5	16	12	7	3
	VeroE6	0.1	16	8	4	4
	VeroE6	0.1	16	6	8	4
	VeroE6	0.1	16	8	8	2
	A459-ACE2	20	16	7	2	1
Total				60	68	30

Supplementary Table 2 Sample preparation overview for whole-cell cryo-ET. Summary of biological replicas, number of milled lamellas, number of acquired and analyzed tomograms from each cell line used in the study.

Infection and plunging	Cells	MOI	Infection hpi	Number of tomograms	Number of tomograms used for analysis
Sample preparation I	A459-ACE2	20	16	5	0
Sample preparation II	A459-ACE2	20	16	3	2
	VeroE6	0.1	16	14	11
	Calu3	5	16	5	2
	VeroE6	0.1	16	13	6
Total				40	21
