# Supplementary material for: SARS-CoV-2 structure and replication characterized by in situ cryo-electron tomography
Source: Nat Commun. 2020 Nov 18;11:5885. doi: 10.1038/s41467-020-19619-7 (PMC7676268; doi:10.1038/s41467-020-19619-7)
Supplement: Supplementary file 3 — Reporting Summary [file 41467_2020_19619_MOESM3_ESM.pdf]

nature research

Corresponding author(s): Petr Chlanda

Last updated by author(s): Oct 20, 2020

Reporting Summary

Nature Research wishes to improve the reproducibility of the work that we publish. This form provides structure for consistency and transparency in reporting. For further information on Nature Research policies, see our Editorial Policies and the Editorial Policy Checklist.

Statistics

For all statistical analyses, confirm that the following items are present in the figure legend, table legend, main text, or Methods section.

n/a	Confirmed	
☐	☒	The exact sample size (*n*) for each experimental group/condition, given as a discrete number and unit of measurement
☐	☒	A statement on whether measurements were taken from distinct samples or whether the same sample was measured repeatedly
☐	☒	The statistical test(s) used AND whether they are one- or two-sided<br>*Only common tests should be described solely by name; describe more complex techniques in the Methods section.*
☒	☐	A description of all covariates tested
☐	☒	A description of any assumptions or corrections, such as tests of normality and adjustment for multiple comparisons
☐	☒	A full description of the statistical parameters including central tendency (e.g. means) or other basic estimates (e.g. regression coefficient) AND variation (e.g. standard deviation) or associated estimates of uncertainty (e.g. confidence intervals)
☐	☒	For null hypothesis testing, the test statistic (e.g. *F*, *t*, *r*) with confidence intervals, effect sizes, degrees of freedom and *P* value noted<br>*Give P values as exact values whenever suitable.*
☒	☐	For Bayesian analysis, information on the choice of priors and Markov chain Monte Carlo settings
☒	☐	For hierarchical and complex designs, identification of the appropriate level for tests and full reporting of outcomes
☒	☐	Estimates of effect sizes (e.g. Cohen's *d*, Pearson's *r*), indicating how they were calculated

Our web collection on statistics for biologists contains articles on many of the points above.

Software and code

Policy information about availability of computer code

Data collection	serialEM
Data analysis	IMOD, MAPS, Amira 2019.3, cryo-CARE, FIJI

For manuscripts utilizing custom algorithms or software that are central to the research but not yet described in published literature, software must be made available to editors and reviewers. We strongly encourage code deposition in a community repository (e.g. GitHub). See the Nature Research guidelines for submitting code & software for further information.

Data

Policy information about availability of data

All manuscripts must include a data availability statement. This statement should provide the following information, where applicable:

- Accession codes, unique identifiers, or web links for publicly available datasets
- A list of figures that have associated raw data
- A description of any restrictions on data availability

Data are available on the EMDB database under the accession numbers: EMD-11863, EMD-11865, EMD-11866, EMD-11867, EMD-11868. The depositions will be released on the next release date: 28.10.2020.

Field-specific reporting

Please select the one below that is the best fit for your research. If you are not sure, read the appropriate sections before making your selection.

- [x] Life sciences
- [ ] Behavioural & social sciences
- [ ] Ecological, evolutionary & environmental sciences

For a reference copy of the document with all sections, see nature.com/documents/nr-reporting-summary-flat.pdf

Life sciences study design

All studies must disclose on these points even when the disclosure is negative.

Sample size	No statistical method was used to determine the sample size. 3 different cell lines infected with SARS-CoV-2 were used for cryo-FIB milling. Out of 68 tomograms, 30 tomograms of best quality were used for the analysis and showed multiple double-membrane vesicles or budding virions. Since morphologically no large variation between the budding/assembled virions were found, we concluded that 30 tomograms are a sufficient representative sample. For whole-cell cryo-ET of released virions, 21 tomograms were used for analysis and for extracting 1570 vRNP subtomograms, a number sufficient to obtain an average with a resolution of 3 nm.
Data exclusions	Cryo-lamellae that showed structure damage by chemical fixation or devitrification were not used for the data analysis.
Replication	Two independent sample preparations (infection, plunge-freezing) were done on A549-ACE2 cells. One sample preparation was done on infected VeroE6 and Calu3 cells (each contained 6-10 electron microscopy grids). Hence 4 biological replicates were used in the study. Detailed table showing the number of cryo-lamellae and tomograms (technical replicas) is provided in the manuscript as supplementary data (Table 1 and 2). Both sample preparations were successful and were used for analyses.
Randomization	The cells were selected for cryo-FIB milling based on a suitable cell position on the grid (center of the square) and therefore a randomized selection is not possible. However, cell seeding and virus infection results in a stochastically distributed infected cells on the EM grids.
Blinding	Blinding was not used in this study but most of the quantifications and measurements were confirmed independently by another person. Since the aim of the study was to describe the morphology of budding viruses and double-membrane vesicles and this project was not hypothesis driven but discovery-motivated we did not implemented blinding into experimental design.

Reporting for specific materials, systems and methods

We require information from authors about some types of materials, experimental systems and methods used in many studies. Here, indicate whether each material, system or method listed is relevant to your study. If you are not sure if a list item applies to your research, read the appropriate section before selecting a response.

Materials & experimental systems

n/a	Involved in the study	
☐	☒	Antibodies
☐	☒	Eukaryotic cell lines
☒	☐	Palaeontology and archaeology
☒	☐	Animals and other organisms
☒	☐	Human research participants
☒	☐	Clinical data
☒	☐	Dual use research of concern

Methods

n/a	Involved in the study	
☒	☐	ChIP-seq
☒	☐	Flow cytometry
☒	☐	MRI-based neuroimaging

Antibodies

Antibodies used	Primary antibody: anti-dsRNA (Scicons, Cat Nr. 10010200), dilution: 1:1000<br>Secondary goat anti-mouse Alexa Fluor 488 antibody (ThermoFisher, A-21131) diluted 1:1000
Validation	Primary antibody:<br>Manufacturer: mAb SCICONS J2 can be used for ELISA, dsRNA-immunoblotting, immunoaffinity chromatography and immunohistochemistry. Specificity reported by manufacturer: The mAb SCICONS J2 recognizes double-stranded RNA (dsRNA) provided that the length of the helix is greater than or equal to 40 bp. dsRNA-recognition is independent of the sequence and nucleotide composition of the antigen. All naturally occurring dsRNAs investigated up to now (40-50 species) as well as poly(I).poly(C) and poly(A).poly(U) have been recognized by SCICONS J2, although in some assays its affinity to poly(I).poly(C) is about 10 times lower than that to other dsRNA antigens.<br>In addition, we have used the antibody staining on uninfected cells and no signal was detected.<br><br>Secondary antibody:<br>Manufacturer: Anti-Mouse secondary antibodies are affinity-purified antibodies with well-characterized specificity for mouse immunoglobulins and are useful in the detection, sorting or purification of its specified target. Secondary antibodies offer increased

versatility enabling users to use many detection systems (e.g. HRP, AP, fluorescence). They can also provide greater sensitivity through signal amplification as multiple secondary antibodies can bind to a single primaryantibody. Most commonly, secondary antibodies are generated by immunizing the host animal with a pooled population of immunoglobulins from the target species and can be further purified and modified (i.e. immunoaffinity chromatography, antibody fragmentation, label conjugation, etc.) to generate highly specific reagents.

Eukaryotic cell lines

Policy information about cell lines

Cell line source(s)	VeroE6, A549, HEK-293T and Calu-3 cells were obtained from ATCC (cat# CRL-1586, CCL-185, CRL-3216 and HTB-55 respectively). A549-ACE2 stable cell line was generated in the Bartenschlager lab.
Authentication	None of the cell lines were authenticated.
Mycoplasma contamination	All cells were tested negative for mycoplasma contamination using the mycoalert kit (Lonza, cat# LT07-118).
Commonly misidentified lines (See ICLAC register)	No commonly misidentified lines were used.

April 2020
