# Supplementary material for: SARS-CoV-2 structure and replication characterized by in situ cryo-electron tomography
Source: Nat Commun. 2020 Nov 18;11:5885. doi: 10.1038/s41467-020-19619-7 (PMC7676268; doi:10.1038/s41467-020-19619-7)
Supplement: Supplementary file 4 — Description of Additional Supplementary Files [file 41467_2020_19619_MOESM4_ESM.pdf]

Description of Additional Supplementary Files

Supplementary Movie 1

Tomogram of DMV with volume rendering and vRNA segmentation. 3D rendering of one tomogram (Fig. 1) of A549-ACE2 cells infected with SARS-CoV-2 at 16 hpi showing a DMV's outer and inner membrane (dark and light green, respectively) as well as manually segmented filaments in the DMV's core, color-coded by total filament length. The tomogram was denoised using cryo-CARE.

Supplementary Movie 2

3D rendering of SARS-CoV-2 virion budding and assembly at the ERGIC membrane (see Fig. 3). 3D rendering of VeroE6 cells infected with SARS-CoV-2 at 16 hpi showing budding events at the ERGIC membrane and intracellular released virions inside the ERGIC lumen. Cellular and viral membranes are shown in green and magenta, respectively. S trimers (yellow) and vRNPs (cyan) are represented as subtomogram averages. The S and vRNP locations correspond to the location in the tomogram, vRNP orientations were randomized. A non-local means filter was applied on the tomogram, and 20 slices were averaged.

Supplementary Movie 3

3D rendering of SARS-CoV-2 virion budding and assembly at the ERGIC membrane (See Supplementary Fig. 6). 3D rendering of VeroE6 cells infected with SARS-CoV-2 at 16 hpi showing intracellular released virions inside the ERGIC lumen. Cellular and viral membranes are shown in green and magenta, respectively. S trimers (yellow) and vRNPs (cyan) are represented as subtomogram averages. The S and vRNP locations correspond to the location in the tomogram, vRNP orientations were randomized. A non-local means filter was applied on the tomogram, and 20 slices were averaged.

Supplementary Movie 4

3D rendering of subtomogram average of S trimer with fitted structures. S trimer subtomogram average (yellow) and the virion envelope (magenta). Fitted structure of the S trimer ectodomain (PDB:6VXX) and the HR2 domain (PDB:2FXP) are shown in black and orange, respectively.

Supplementary Movie 5

3D rendering of subtomogram average of vRNP. vRNP complex rotated around the long and short axis.
