# Supplementary material for: SARS-CoV-2 structure and replication characterized by in situ cryo-electron tomography
Source: Nat Commun. 2020 Nov 18;11:5885. doi: 10.1038/s41467-020-19619-7 (PMC7676268; doi:10.1038/s41467-020-19619-7)
Supplement: Supplementary file 2 — Peer Review File [file 41467_2020_19619_MOESM2_ESM.pdf]

Reviewer #1 (Remarks to the Author):

This manuscript reports a study making use of CryoET and FIB milling to study the viral infection/replication process of SARS-CoV-2 in-situ. Given the ongoing pandemic, clearly this study is quite timely, and likely to attract broad interest. As primarily an observational study, rather than testing actual hypotheses related to the virus, it would likely be used primarily for hypothesis generation in followup studies. As such I would say many of the observations being made are quite interesting, and indeed could push further research in this area. The methodology being employed is cutting-edge, making use of cryo-FIB milling to perform imaging within cryopreserved cells. While chemical fixation was used in advance of cryopreparation as a safety measure, this is almost unavoidable due to a lack of CryoEM facilities supporting higher BSL research. All of the procedures followed are fairly standard in the field and seem to have been performed well. Data quality is excellent. I believe the risk that the fixation process fundamentally altered the results of the study are reasonably low, and as this process is freely acknowledged, subsequent research can take this point into account when considering conclusions.

Overall I find this to be an impactful, skillfully performed series of experiments worthy of rapid publication, and of sufficiently broad interest for this journal. A few of the observations may be questionable as they are based on a single observed instance or on a very small number of instances, but again, this is quite clear in the manuscript, and claims do not seem to be exaggerated. A limited number of instances is somewhat inherent to this methodology at the current stage of development. My only minor suggestions for improving the manuscript would be to do a better job of defining acronyms used throughout the manuscript. While a CryoET virologist is unlikely to have any difficulty with it, a broader audience will find these challenging. Other than this very minor point I would support rapid publication of this manuscript.

Reviewer #2 (Remarks to the Author):

In this manuscript Klein et al used cryo electron tomography to characterise SARS-CoV-2 in the context of three different cell lines and at different point of the virus replication cycle. They characterised the double membrane vesicles (DMV), the compartment associated with viral replication, in terms of size, content and interactions. They further described virion budding sites, intracellular virions with their S trimers on membranes and vRNP. Finally, they described the attachment of virions to the cell lines they used.

Taken together the authors presented a substantial amount of data but this is also the weakness of this manuscript. There is a lot of data but it is not put in an order that is rational and easy to follow. One example, they are using for their segmentation in Fig 3 the averages they got for the S trimer and vRNP that are shown and explained in Fig 4 and 5 respectively. The other major problem with lacking a focus is that non of the concepts and analysis go deep and all stay in the descriptive level. And by now there are other publications with better quality. For example DMV and their content were recently published in Science together with the portal that allows transport of the RNA (Wolff et al). A better resolution and analysis of the pre-fusion conformation of S trimer with the flexibility relative to the membrane was also recently published in both Science and Nature (Turonuva et al and Ke et al). Thus, taken together the added value of this manuscript is very limited.

Below are some specific comments and questions:
- Pg. 6 line 2 the reference to Fig S4 is wrong here as this figure show the tilted spikes
- Why the DMV measurements are coming from such a small data set?
- All the segmentation done in this manuscript was manual, which is subjective. The gold standard in the field is more automated ones based on neural networks.
- Pg. 7 line 22 onwards. The discussion about DMV fusion is not convincing based on the provided data.

- The authors should provide the FSC curves and resolution of their averages (S trimer and vRNP).
- The vRNP average is of very low resolution and lack clear features, yet the authors attempt to interpret the average (pg. 17 line 17 onwards). All this description is very speculative. They refer to ‘beads on string’ that I cannot see.
- What limit the resolution of the vRNP? with 1570 particles I would expected higher resolution. The authors should comment on that.
- In Fig 3 and 4 it says that the ‘vRNP orientations were randomised’, why is that? they should have had the orientation information from their averaging.
- Fig 3 a. The segmentation is not exactly the same field of view as the slice provided. The authors should either provide the same field of view or provide scale bar to the segmentation. The same for Fig 4 (a) and (b).
- Pg 13 line 17 Fig S5 is about the vaults protein complex and not the S protein.
- A major part of the discussion is speculative in particular pg. 20 line 11 onwards.

REVIEWER COMMENTS

Reviewer #1 (Remarks to the Author):

I really had no negative comments aside from the abundance of acronyms in the initial submission.

Regarding the second reviewer's comments. I would take issue with the statement that deep learning based annotation is the gold standard, and should be used instead of human annotation. While the developers of the current generation of deep-learning based annotation tools have a goal of producing something which is reliable, I would not say any of these algorithms are presently better than a human, they are simply faster and more convenient. Indeed, the first of these algorithms requires human annotations for training. In any case, I do not find any issues with the annotation performed in this manuscript.

On the topic of the resolution of the subtomogram average, the difference between 13 Å resolution and 30 Å resolution is not slight, it is vast. However, I did not raise this complaint, as there are several reasonable explanations for the 30 Å resolution limit, including the fixation process and the use of simple phase-flipping corrections in IMOD, which for thick specimens often fail to achieve resolutions beyond 20 - 30 Å. The manuscripts now published with subnanometer resolution subtomogram averages all make use of far more advanced methodologies than used in this manuscript. While it is possible that better resolution could be achieved with different software, with fixation, I question how useful this would really be, even if there isn't another inherent limit.

In any case, I still feel this manuscript is worthy of publication, and will leave comments on the second reviewer's other points to him/her.

Response to the reviewer 1:

Thank you for your positive comments. We share the opinion about deep-learning algorithms. We attempted to use deep-learning-based automated segmentation that is implemented in Amira but it did not yield segmentations that could be used for filament length measurements. We appreciate that reviewer 1 checked the additional data requested by reviewer 2. We agree that the resolution obtained is much lower in comparison to the recently published vRNP structure and we agree that this is likely due to the lower number of used particles and also due to the use of simple phase-flipping corrections in IMOD. While the vRNP structure could be improved using for example 3D CTF correction, we believe that for further attemps to obtain a higher resolution structure of the vRNP would be better to use an un-fixed sample and therefore a BSL-2 coronavirus would be a better choice for that.

Reviewer #1 (Remarks to the Author):

I really had no negative comments aside from the abundance of acronyms in the initial submission.

Regarding the second reviewer's comments. I would take issue with the statement that deep learning based annotation is the gold standard, and should be used instead of human annotation. While the developers of the current generation of deep-learning based annotation tools have a goal of producing something which is reliable, I would not say any of these algorithms are presently better than a human, they are simply faster and more convenient. Indeed, the first of these algorithms requires human annotations for training. In any case, I do not find any issues with the annotation performed in this manuscript.

On the topic of the resolution of the subtomogram average, the difference between 13 Å resolution and 30 Å resolution is not slight, it is vast. However, I did not raise this complaint, as there are several reasonable explanations for the 30 Å resolution limit, including the fixation process and the use of simple phase-flipping corrections in IMOD, which for thick specimens often fail to achieve resolutions beyond 20 - 30 Å. The manuscripts now published with subnanometer resolution subtomogram averages all make use of far more advanced methodologies than used in this manuscript. While it is possible that better resolution could be achieved with different software, with fixation, I question how useful this would really be, even if there isn't another inherent limit.

In any case, I still feel this manuscript is worthy of publication, and will leave comments on the second reviewer's other points to him/her.

2REVIEWER COMMENTS

Reviewer #1 (Remarks to the Author):

This manuscript reports a study making use of CryoET and FIB milling to study the viral infection/replication process of SARS-CoV-2 in-situ. Given the ongoing pandemic, clearly this study is quite timely, and likely to attract broad interest. As primarily an observational study, rather than testing actual hypotheses related to the virus, it would likely be used primarily for hypothesis generation in followup studies. As such I would say many of the observations being made are quite interesting, and indeed could push further research in this area. The methodology being employed is cutting-edge, making use of cryo-FIB milling to perform imaging within cryopreserved cells. While chemical fixation was used in advance of cryopreparation as a safety measure, this is almost unavoidable due to a lack of CryoEM facilities supporting higher BSL research. All of the procedures followed are fairly standard in the field and seem to have been performed well. Data quality is excellent. I believe the risk that the fixation process fundamentally altered the results of the study are reasonably low, and as this process is freely acknowledged, subsequent research can take this point into account when considering conclusions.

Overall I find this to be an impactful, skillfully performed series of experiments worthy of rapid publication, and of sufficiently broad interest for this journal. A few of the observations may be questionable as they are based on a single observed instance or on a very small number of instances, but again, this is quite clear in the manuscript, and claims do not seem to be exaggerated. A limited number of instances is somewhat inherent to this methodology at the current stage of development. My only minor suggestions for improving the manuscript would be to do a better job of defining acronyms used throughout the manuscript. While a CryoET virologist is unlikely to have any difficulty with it, a broader audience will find these challenging. Other than this very minor point I would support rapid publication of this manuscript.

Authors response: Thank you very much for this positive review. We agree with the reviewer that in the manuscript many acronyms were used, which is inherent to the used methods. We made sure to introduce each acronym the first time it was used in the text. In addition, we removed the introduction of abbreviations which were used only sparsely in the whole text, namely: single-stranded positive-sense (+RNA), viral RNA (vRNA), nonstructural protein (nsp), cryo-focused ion beam (cryo-FIB), Subtomogram averaging (STA).

Reviewer #2 (Remarks to the Author):

In this manuscript Klein et al used cryo electron tomography to characterise SARS-CoV-2 in the context of three different cell lines and at different point of the virus replication cycle. They characterised the double membrane vesicles (DMV), the compartment associated with viral replication, in terms of size, content and interactions. They further described virion budding sites, intracellular virions with their S trimers on membranes and vRNP. Finally, they described the attachment of virions to the cell lines they used.

Taken together the authors presented a substantial amount of data but this is also the

weakness of this manuscript. There is a lot of data but it is not put in an order that is rational and easy to follow. One example, they are using for their segmentation in Fig 3 the averages they got for the S trimer and vRNP that are shown and explained in Fig 4 and 5 respectively.

Authors response: We want to thank the reviewer for the general positive comments and the acknowledgement of the substantial amount of provided data. We aimed to describe the replication cycle of the virus starting with RNA replication (DMVs) and virus assembly inside the cell followed by observations on extracellular virions (S trimer pre- and post-fusion, vRNP structure). Thus, the logic of the order follows the virus replication cycle from RNA→ proteins → virus.

The other major problem with lacking a focus is that non of the concepts and analysis go deep and all stay in the descriptive level. And by now there are other publications with better quality. For example DMV and their content were recently published in Science together with the portal that allows transport of the RNA (Wolff et al).

Authors response: We agree with the author that the recent study by Wolff *et. al.* is very valuable, and especially the identification of a pore is of high importance to the field. However, most of the data provided by Wolff *et al.* is on cells infected with murine hepatitis virus (MHV). Our study using cryo-FIB milling and cryo-ET delivers additional data on SARS-CoV-2 coronaviruses complementing the study by Wolff *et. al.* In addition to Wolff *et al.* we provide insight such as the detailed analysis of the viral RNA in DMVs, viral assembly sites at the ERGIC and the structural analysis of intracellular virions.

A better resolution and analysis of the pre-fusion conformation of S trimer with the flexibility relative to the membrane was also recently published in both Science and Nature (Turonuva et al and Ke et al). Thus, taken together the added value of this manuscript is very limited.

Authors response: The two recent publications are of great value and analyzed the structural flexibility of the S trimers in great detail. The studies used released virions in the vicinity of the cell grown on an EM grid (Ke *et al.*) or purified virus particles (Turonova *et al.*). Our study brings additional value by examining the S trimer structure on intracellular virus particles in the native cellular context, validating that the S flexibility is not an artefact of the virus purification or blotting forces exerted on released virions in the vicinity of the cell.

Below are some specific comments and questions:

- Pg. 6 line 2 the reference to Fig S4 is wrong here as this figure show the tilted spikes

Authors response: Thank you very much for finding this wrong reference. We updated the reference *(p6, line 29).*

- Why the DMV measurements are coming from such a small data set?

Authors response: We measured the DMV diameter for all three cell lines. In total we measured 20 DMVs for VeroE6 cells, 14 DMVs for A549 cells and 3 DMVs for Calu3 cells, as shown in Fig. 1C. In the main text, we reported the results for VeroE6 only instead of the

average and standard deviation of the complete data set. This was an unintended mistake and we want to thank the reviewer for point this out. We updated the main text to include all 38 measurements. The average diameter changed from 336 ± 50 nm to 338 ± 56 nm *(p4, lines 1 – 2)*.

- All the segmentation done in this manuscript was manual, which is subjective. The gold standard in the field is more automated ones based on neural networks.

Authors response: Thank you very much for this comment. Prior to segmentation we have denoised the data using a deep learning denoising algorithm (cryo-CARE) to segment the RNA inside the DMVs, this by itself is one of the least biased denoising approach currently available. We used a semi-automated segmentation approach. The initial segmentation was performed in an automated fashion using the Top-Hat segmentation approach implemented in Amira. This intermediate result was manually corrected by removing segmented noise and manually correct areas which were not correctly segmented. This process was chosen to minimize subject segmentation. To our knowledge, a fully automated segmentation based on neural networks is to date only possible for electron microscopy data of samples which were chemically fixed and stained, resulting in a contrast rich image. Cryo-EM data shows a much lower signal-to-noise ratio, preventing fully automated segmentation. We updated the materials and methods section for tomogram reconstruction:

(p14, lines 7 – 10) This initial segmentation was manually refined by removing segmented sections derived from noise and by manually adding segmentations of membranes where automated segmentation failed.

- Pg. 7 line 22 onwards. The discussion about DMV fusion is not convincing based on the provided data.

Authors response: We agree that the proposed mechanism of membrane fusion between DMVs is different from the classical membrane fusion driven by fusion proteins. Importantly although DMV fusion has been reported before, no mechanism has been proposed. Since membrane fusion proteins such as influenza A virus hemagglutinin or SNAREs bring the two membranes in close proximity overcoming the electrostatic repulsion of the membrane surfaces, it seems plausible that membrane stacking could lead membrane fusion. We modified the following section to:

(p5, line 13 – 14) Tight membrane apposition might lead to lipid mixing between the stacked bilayers followed by membrane fusion.

- The authors should provide the FSC curves and resolution of their averages (S trimer and vRNP).

Authors response:

We calculated the FSC curve of the vRNP and estimated the resolution to be 33 Å based on the 0.5 criterion and added the information to the manuscript *(p.9, lines 3 – 4)*. The FSC calculated of the S trimer was 90 Å (0.5 criterion). We would like to stress that only 219 spikes

were used, and these data come from cryo-ET performed on lamella. The FSC curves are shown in Supplementary Fig. 7 and 11. We updated the Methods section to include resolution estimation analysis *(p14, line 28 – p. 15, line 6)*.

- The vRNP average is of very low resolution and lack clear features, yet the authors attempt to interpret the average (pg. 17 line 17 onwards). All this description is very speculative. They refer to 'beads on string' that I cannot see.

Authors response: A study published during the revision by Yao *et al.* reported a vRNP STA structure with a similar morphology at a resolution of 1.31 nm from 18,500 individual vRNPs which supports the here reported vRNPs structure as individual units with a characteristic shape (doi.org/10.1016/j.cell.2020.09.018). By identification of these individual units, it is reasonable to conclude that the complete viral genome consists of one viral RNA which is bound to several vRNPs complexes connected by viral RNA. This arrangement can be described as 'beads on string'. We added the reference to the preprint of Yao *et al.:*

(p9, lines 10 – 12). A recent study by Yao et. al reported a SARS-CoV-2 vRNP structure with a similar morphology to the here presented STA.

- What limit the resolution of the vRNP? with 1570 particles I would expected higher resolution. The authors should comment on that.

Authors response: The resolution of the vRNP complex is likely limited by the flexible arrangements and different number of N proteins within the complex. The estimated resolution of 33 Å was achieved by subtomogram averaging using 1570 individual vRNP units. In the recent study by Yao *et al.* 18,500 individual units were used, which only slightly increased the resolution to 13 Å, which is another evidence that the structure is quite heterogenous and it will be challenging to resolve the vRNP at higher resolution.

- In Fig 3 and 4 it says that the 'vRNP orientations were randomised', why is that? they should have had the orientation information from their averaging.

Authors response: The vRNP STA was calculated based on tomograms of extracellular virions, as described in the text, and not from tomograms of cryo-FIB milled samples. To indicate positions of vRNPs inside the budding virions, we placed the vRNP averages into the rendering based on the position of the vRNP densities in the tomogram, however, the orientation could not always be unequivocally determined and therefore the orientation of the vRNP average was randomized in the renderings shown in Fig. 3 and 4.

- Fig 3 a. The segmentation is not exactly the same field of view as the slice provided. The authors should either provide the same field of view or provide scale bar to the segmentation.The same for Fig 4 (a) and (b).

Authors response: Thank you very much for this suggestion. We added scalebars to Fig. 3, Fig. 4b and Supplementary Fig. 6a.

- Pg 13 line 17 Fig S5 is about the vaults protein complex and not the S protein.

Authors response: Thank you very much for pointing out this mistake. We removed the wrong reference *(p6, line 29)*.

- A major part of the discussion is speculative in particular pg. 20 line 11 onwards.

Authors response: Thank you very much for this comment. We removed the speculative section *(p10, line 3)*.
